# GRASP: a computational platform for building kinetic models of cellular metabolism

**DOI:** 10.1093/bioadv/vbac066

**Published:** 2022-09-27

**Authors:** Marta R A Matos, Pedro A Saa, Nicholas Cowie, Svetlana Volkova, Marina de Leeuw, Lars K Nielsen

**Affiliations:** Novo Nordisk Foundation Center for Biosustainability, Technical University of Denmark, 2800 Kgs. Lyngby, Denmark; Department of Chemical and Bioprocess Engineering, School of Engineering, Pontifical Catholic University of Chile, Santiago 7820436, Chile; Institute for Mathematical and Computational Engineering, Pontifical Catholic University of Chile, Santiago 7820436, Chile; Novo Nordisk Foundation Center for Biosustainability, Technical University of Denmark, 2800 Kgs. Lyngby, Denmark; Novo Nordisk Foundation Center for Biosustainability, Technical University of Denmark, 2800 Kgs. Lyngby, Denmark; Novo Nordisk Foundation Center for Biosustainability, Technical University of Denmark, 2800 Kgs. Lyngby, Denmark; Novo Nordisk Foundation Center for Biosustainability, Technical University of Denmark, 2800 Kgs. Lyngby, Denmark; Australian Institute for Bioengineering and Nanotechnology, The University of Queensland, St Lucia, QLD 4072, Australia

## Abstract

**Summary:**

Kinetic models of metabolism are crucial to understand the inner workings of cell metabolism. By taking into account enzyme regulation, detailed kinetic models can provide accurate predictions of metabolic fluxes. Comprehensive consideration of kinetic regulation requires highly parameterized non-linear models, which are challenging to build and fit using available modelling tools. Here, we present a computational package implementing the GRASP framework for building detailed kinetic models of cellular metabolism. By defining the mechanisms of enzyme regulation and a reference state described by reaction fluxes and their corresponding Gibbs free energy ranges, GRASP can efficiently sample an arbitrarily large population of thermodynamically feasible kinetic models. If additional experimental data are available (fluxes, enzyme and metabolite concentrations), these can be integrated to generate models that closely reproduce these observations using an approximate Bayesian computation fitting framework. Within the same framework, model selection tasks can be readily performed.

**Availability and implementation:**

GRASP is implemented as an open-source package in the MATLAB environment. The software runs in Windows, macOS and Linux, is documented (graspk.readthedocs.io) and unit-tested. GRASP is freely available at github.com/biosustain/GRASP.

**Supplementary information:**

Supplementary data are available at *Bioinformatics Advances* online.

## 1. Introduction

Kinetic models offer a unique and comprehensive description of cell metabolism. By taking into account enzyme regulation, they can predict how reaction fluxes and metabolite concentrations respond to genetic and environmental perturbations. However, the construction of such models requires large amounts of data to fit a multitude of parameters. These are typically either measured *in vitro* or inferred from *in vivo* fluxomics/metabolomics data (Saa and Nielsen, 2017). The former being hard to obtain for less well-studied pathways/organisms, and the latter being challenging to calibrate due to their inherent sloppy sensitivities (Gutenkunst *et al.*, 2007).

We previously introduced the GRASP framework, which leverages a single steady-state flux distribution and the corresponding reaction Gibbs free energy profile to generate a population of thermodynamically consistent models (Saa and Nielsen, 2015, 2016). The resulting population of models is amenable for a variety of analyses such as metabolic control analysis (MCA), whereby enzymes exerting the highest flux control can be identified. This is essential for metabolic engineering and, more fundamentally, for understanding flux regulation. GRASP was later extended for performing parameter inference and model selection using a rejection sampler within an approximate Bayesian computation (ABC) setting (Saa and Nielsen, 2016). The latter enables the integration of several omics datasets measured under different steady-state conditions, which increases prediction fidelity and reduces parameter uncertainty.

While different tools and algorithms have been proposed for calibrating dynamic models in general (Villaverde *et al.*, 2022), and fitting kinetic models in particular (Chakrabarti *et al.*, 2013; Gopalakrishnan *et al.*, 2020), very few have led to software packages readily usable by the metabolic modelling community. To the best of our knowledge, there is currently a lack of fully contained computational packages enabling: (i) specification and automatic construction of feasible kinetic models of metabolism, (ii) exploration of their feasible kinetic behaviour, (iii) Bayesian-based parameter fitting and model selection and (iv) simulation of dynamic and systems properties (e.g. flux control coefficients). Here, we present the implementation of GRASP in MATLAB for tackling these limitations.

## 2. Computational implementation

GRASP is implemented as a library of MATLAB functions. The software runs in Windows, macOS and Linux, is documented (graspk.readthedocs.io) and unit-tested. GRASP makes also use of the Bioinformatics, Optimization and Parallel Computing Toolboxes from MATLAB, albeit the latter is not strictly necessary for single-core computations. External dependencies can be avoided if the user chooses linear and nonlinear optimization solvers included in MATLAB, although additional alternatives are also supported [Gurobi (Gurobi Optimization, LLC, 2022)] for LP and MILP problems, and NLOPT (Johnson, 2021 for non-linear problems). For sampling large model ensembles, GRASP can take advantage of multiple cores with an almost linear scaling when the Parallel Computing Toolbox is available. For increasing software usability, two small Python packages are provided for easing the construction of the input file for GRASP (see set_up_grasp_models at github.com/biosustain/set_up_grasp_models), and for visualizing MCA and simulation results. The use of additional Python packages is optional and is intended to facilitate the previous tasks to the user if he/she decides to use them. Finally, the GRASP package is released under the GNU General Public License. We welcome community contributions to the GitHub repository where it is hosted.

## 3. GRASP workflow

**Fig. 1.**
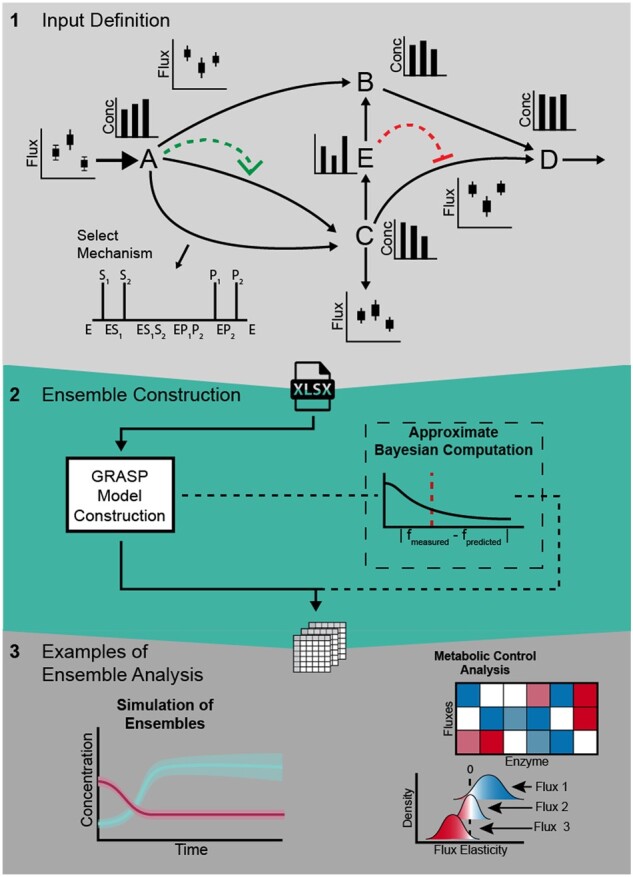
Workflow for building kinetic models with GRASP. (1) Input information required to construct a kinetic model ensemble: the stoichiometric matrix, flux and metabolite concentration ranges, catalytic mechanisms and allosteric interactions (arrow head and arrow bar, respectively). (2) The GRASP framework generates a population (ensemble) of thermodynamically feasible kinetic models anchored at the reference state. If data for more than one steady state is available, ABC is used to build a population of models consistent with the reference state and the additional experimental data. (3) The resulting model ensemble can be employed to compute a distribution of metabolic fluxes and metabolite control coefficients under different conditions, as well as to simulate dynamic perturbations from the initial state by altering enzyme concentrations and/or metabolite concentrations

The main three steps to building a kinetic model population are (i) input preparation, (ii) parameter simulation and fitting and (iii) *a posteriori* analysis (Fig. 1, refer to the Supplementary Information for the details of each step). In the first step, the reference state and model information are defined including catalytic and allosteric mechanisms. The reference state is defined a steady-state flux distribution and the corresponding Gibbs free energy profile for the model reactions. If omics datasets (e.g. metabolomics, proteomics and fluxomics) are available for additional steady-state conditions, these can be included for building a population of models that fits them. Additional simulation parameters such as number of models, simulation mode, error tolerance, solver, among others, must be defined in this phase.

The second step samples a population of feasible kinetic models according to the chosen simulation mode. GRASP currently supports two sampling modes: (i) prior sampling at the reference state (GRASP) and (ii) ABC-rejection sampling consistent with various experimental conditions (rejection). The latter mode can also be employed for simultaneously performing model selection, enabling for example the identification of allosteric interactions supported by the data (Saa and Nielsen, 2016).

The final step consists of the quantitative analysis of the model population (ensemble). This population is encoded as a MATLAB structure, which can be readily used to perform MCA, as well as time-course simulations using GRASP functions. In addition, the user can export individual models to SBML format. To increase the usability of GRASP and facilitate the visualization of MCA and simulation results, an additional Python package was developed along with illustrative Jupyter notebooks, which are available in the GRASP repository.

## 4. Case study: control analysis of the central carbon metabolism of *Pseudomonas putida*

**Fig. 2.**
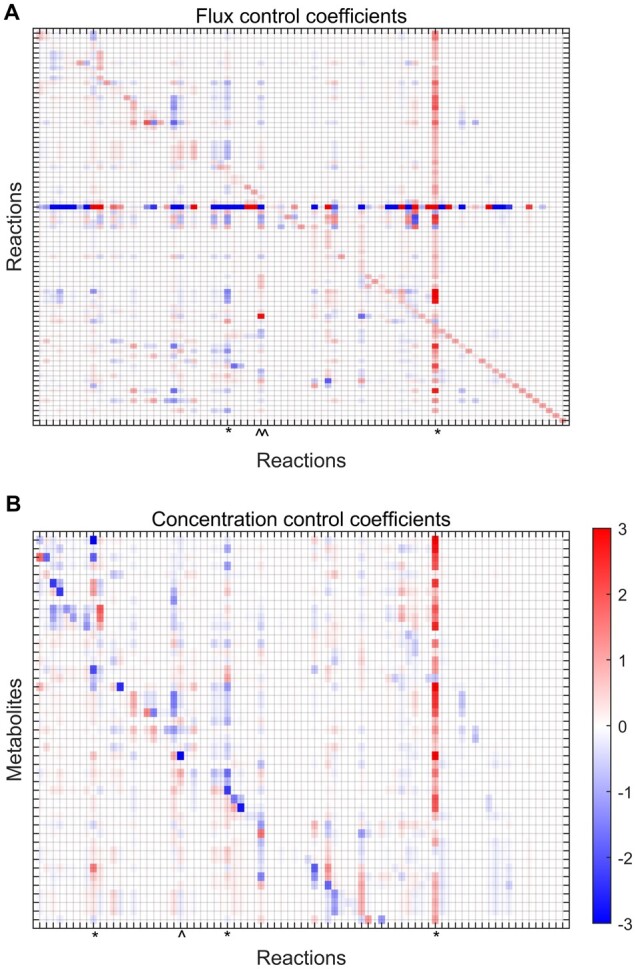
Metabolic control structure of the central carbon metabolism of *Pseudomonas putida*. The control exerted by each reaction to each reaction flux (**A**) and metabolite concentration (**B**) can be explored by browsing the heatmap vertically. Results indicate that both the flux and concentration control is distributed and exerted by the upper glycolysis (around glucose and fructose 6-phosphate nodes through the glucose transporter, fructose bisphosphatase and fructose-bisphosphate aldolase reactions) and the entry point to the TCA cycle (through the malic enzyme, malate dehydrogenase, pyruvate dehydrogenase and pyruvate kinase, among others). High-control reactions of the upper glycolysis and entry points to the TCA cycle are marked with (*) and (∧), respectively

In order to illustrate the use and capabilities of GRASP for building and analysing kinetic models of metabolism, we prepared a demonstration of its performance in a complex system and perform MCA on the resulting ensemble (Fig. 2). A step-by-step tutorial for the simulation of this model as well as the model specification file employed for model setup can be found in the tutorial 05 file (github.com/biosustain/GRASP/tree/main/tutorials). Four additional tutorials describing model structures of increasing complexity are also available in the GitHub repository for GRASP.

Inspired by a recent kinetic model of the central carbon metabolism of *Pseudomonas putida* (Kozaeva *et al.*, 2021), we have formulated and built a model with 79 reactions, where 27 of those are either exchange reactions (to allow metabolites to be exchanged with the cell’s environment) or re-generation reactions to re-convert certain metabolites, e.g. NAD/NADH, NADP/NADPH and ADP/ATP. These reactions are modelled by simple mass action rate laws, whereas the remaining 52 reactions were decomposed into elementary reaction steps each modelled using mass action yielding detailed kinetic parameterizations (Saa and Nielsen, 2017). The model also includes two isoenzymes, PYK1 and PYK2, where the flux distribution between is unknown, and seven promiscuous enzymes. Lastly, the model includes several competitive inhibitors and allosteric effectors.

Sampling computation can last anywhere from 30 min to 1 h on a conventional single-core laptop computer. Once the sampling is carried out, MCA can be performed on the model. Here, this analysis revealed that both the flux and concentration control is distributed and exerted in the upper glycolysis (around the fructose 6-phosphate node through the fructose bisphosphatase and fructose-bisphosphate aldolase reactions), and the pyruvate–malate axis in the TCA cycle (through the malic enzyme, malate dehydrogenase, pyruvate dehydrogenase and pyruvate kinase, among others).

## 4. Conclusion

The GRASP framework implementation enables the construction and simulation of thermodynamically feasible and detailed kinetic models of cellular metabolism. To the best of our knowledge, this is the first open-source well-documented MATLAB package of its type that enables building, analysing and sharing detailed kinetic models consistent with thermodynamic and experimental data. By enabling the prediction of metabolic fluxes and control coefficients from kinetic models, we expect GRASP to improve the understanding of biologically relevant reactions and pathways, which are often under complex metabolic regulation. For this task, GRASP provides a robust computational suite that can be further expanded as more advanced kinetic modelling tools become available, particularly for parameter sampling and fitting. Community contributions will not only be welcome, but they will be critical to increase current computational capabilities.

## Funding

This work was supported by the Novo Nordisk Foundation [NNF14OC0009473 and NNF20CC0035580]; ANID through Fondecyt de Iniciación [11190871 to P.A.S.]; and a Copenhagen Bioscience PhD fellowship [Novo Nordisk Foundation, NNF17CC0026768 to S.V.].

*Conflict of Interest*: none declared.

## Supplementary Material

vbac066_Supplementary_Data
