# Supplementary material for: GRASP: a computational platform for building kinetic models of cellular metabolism
Source: Bioinform Adv. 2022 Sep 27;2(1):vbac066. doi: 10.1093/bioadv/vbac066 (PMC9710608; doi:10.1093/bioadv/vbac066)
Supplement: vbac066_Supplementary_Data [file vbac066_supplementary_data.pdf]

Supporting Information

Enzyme kinetics within our GRASP: A computational platform for building kinetic models of cellular metabolism

Marta R. A. Matos, Pedro A. Saa, Nicholas Cowie,
Svetlana Volkova, Marina de Leeuw and Lars K. Nielsen

September 12, 2022

Contents

1 The GRASP framework **2**

2 Computational workflow **2**

2.1 Input preparation 3

2.2 Parameter simulation and fitting 3

2.3 *A posteriori* analysis 4

3 Optimization, sampling and numerical methods **4**

3.1 Modified TMFA 4

3.2 Flux and thermodynamic sampling 6

3.3 MCA for promiscuous enzymes 6

1 The GRASP framework

Kinetic models are described by a set of Ordinary Differential Equations (ODEs) representing the dynamic mass balances for the reacting species in the system,

$$\dot{\mathbf{x}} = \mathbf{S} \cdot \mathbf{r}(\mathbf{x}, \mathbf{e}, \mathbf{k}) \quad (1)$$

$$\mathbf{x}(0) = \mathbf{x}_0 \quad (2)$$

where $\mathbf{S}$ and $\mathbf{r}$ denote the stoichiometric matrix and the vector of reaction rates, respectively, $\mathbf{x}_0$ is the initial condition for the metabolite concentrations, $\mathbf{k}$ is a vector of kinetic parameters, and $\mathbf{e}$ denotes enzyme concentrations. Of special interest in metabolic systems is the determination of the steady-state of Eq. (1), i.e., when $\dot{\mathbf{x}} = \mathbf{0}$. This state defines a stable metabolic state, which can be readily parameterized using previous approaches [**?**, Saa and Nielsen(2015)Saa and Nielsen]. Each reaction rate r is computed as $r = e \cdot v(\mathbf{x}, \mathbf{k})$; where e denotes the corresponding enzyme concentration and v describes the enzyme activity. GRASP uses the generalized MWC model [**?**, **?**, **?**, **?**] to model the reaction flux through an oligomeric enzyme as the product of a catalytic function (Φ_{cat}) and a regulatory function (Ψ_{reg}):

$$v = \Phi_{\text{cat}} \cdot \Psi_{\text{reg}} \quad (3)$$

where:

$$\Phi_{\text{cat}} = n \cdot v_{\text{R}} \quad (4)$$

$$\Psi_{\text{reg}} = \frac{1 + (v_{\text{T}}/v_{\text{R}})\,Q}{1 + Q} \quad (5)$$

v_{R} is the flux catalyzed by the active (R) state of the enzyme, and v_{T} is the flux catalyzed by the tense (T) state of the enzyme. Q is a function that defines the ratio of enzyme in the R and T state. To parameterize Φ_{cat}, GRASP decomposes each reaction into elementary reactions modeled by mass-action kinetics. By defining a reference state, enzyme and metabolite concentrations as well as the elementary rate constants are scaled appropriately. The scaled elementary rate constant values are then calculated based on sampled elementary reversibilities and relative enzyme state concentrations, thereby ensuring thermodynamic consistency [Saa and Nielsen(2015)Saa and Nielsen]. If either $\boldsymbol{v}^{\text{ref}}$ and/or $\Delta_{\text{r}}\boldsymbol{G}^{\text{ref}}$ cannot be uniquely determined from the input data (see next section), a general Hit-And-Run sampler [**?**] is employed to capture this uncertainty while ensuring kinetic consistency. Execution of this step only requires reaction directionalities and the definition of a sampling function by the user (uniform or normal are supported). Finally, integration of additional omics datasets for different conditions can be readily performed for improving parameter inference using a rejection-ABC sampler [Saa and Nielsen(2016)Saa and Nielsen].

2 Computational workflow

GRASP is implemented as a library of MATLAB functions that only needs to be added to the path before running. The following toolboxes are required to run GRASP: Bioinformatics, Optimization and Parallel Computing Toolbox. External dependencies can be avoided if the user chooses to use the linear (`intlinprog`) and nonlinear (`fmincon`) optimization solvers included in Matlab. However, Gurobi [Gurobi Optimization, LLC(2021)Gurobi Optimization, LLC] is also supported as an LP/MILP solver, as well as NLOPT [Steven G. Johnson(2021)Steven G. Johnson] as a nonlinear optimization solver. The main three steps to build a kinetic model are input preparation, parameter simulation and fitting, and *a posteriori* analysis.

2.1 Input preparation

GRASP uses a spreadsheet (`.xlsx` file) to specify the reference state and model information, namely: reaction stoichiometries, standard Gibbs energies for each reaction $\Delta_r \boldsymbol{G}^{'0}$ (kJ/mol) (typically obtained from *eQuilibrator* [?], absolute metabolite concentrations (mol/L) and reaction fluxes (mmol/L/h) at the reference state, along with enzyme mechanisms and regulation. Enzyme mechanisms are defined following [?], where the binding order of each substrate and release order of each product, as well as the binding/release of inhibitors (competitive/uncompetitive/mixed) or activators are specified. Each mechanism must be provided as a separate `.txt` file with a specific format. The most common mechanisms are already provided, whereas customized ones can be defined by the user. Allosteric effectors are not included in the enzyme mechanism, instead they are specified in the input file.

When omics datasets are available to generate parameter sets that fit multiple conditions, proteomic and metabolomic data must be provided for each condition as relative values to the reference state. In contrast, absolute fluxes must be provided for each condition. Additionally, conservation relations (i.e., linear constraints) can be defined for groups of pool metabolites (e.g., NAD/NADH) for the experimental conditions.

Since building the input file can be rather cumbersome - especially for larger models -, a small python package was developed to simplify this task (see `https://github.com/biosustain/set_up_grasp_models`). It takes a text file with the list of reactions in the system and `.csv` or `.xlsx` files with the fluxomics and metabolomics data to draft the GRASP input file. If BiGG IDs [?] are used in the reaction specifications, the *eQuilibrator* API [?] can be used to obtain $\Delta_r G^{'0}$ values automatically. This package also provides a more intuitive way of specifying reaction mechanisms based on elementary reactions as well as basic checks on the validity of the `.xlsx` input file.

2.2 Parameter simulation and fitting

GRASP generates a population of models that fit the data using the ensemble modeling framework for defining convenient sampling functions (priors). For each catalytic function of each enzyme, GRASP samples enzyme intermediate abundances and microscopic reversibilities from Dirichlet distributions, which ensures mass conservation and thermodynamic consistency. Particularly in the case of the reversibilities, the $\Delta_r G^{\text{ref}}$ value is required to scale them appropriately and must be globally valid, i.e., compatible with other $\Delta_r G^{\text{ref}}$ and consistent with the respective reaction fluxes. To ensure this is the case, a modified version of Thermodynamic-based Metabolic Flux Analysis [?] is implemented to determine feasible $\Delta_r G^{\text{ref}}$ ranges consistent with the directionalities of the reference flux distribution and that ensures the sum of $\Delta_r G^{\text{ref}}$ of all reactions involved in closed loops are zero. GRASP can also take advantage of incomplete fluxomics datasets, as long as all the fluxes can be determined and they are consistent with the defined directionalities. Lastly, ratio inequality constraints (linear in logarithmic space) on metabolite concentrations are also supported for tightening the feasible reference state.

If experimental data are provided for additional steady state conditions, GRASP iteratively proposes random parameters from the defined priors and simulates the experimental data. If the parameterized model reproduces the observed data within some user-defined tolerance level, the parameter set is accepted, otherwise rejected. The final result consists of a population of models (ABC posterior distribution) that reproduces the data within a certain tolerance. Notably, the same procedure can be employed to perform model selection, allowing the user to, e.g., find which allosteric interactions are more likely given the data.

GRASP supports the modeling of promiscuous enzymes by specifying the correct mechanism, as well as isoenzymes. Moreover, it enables the specification of metabolite pool constraints, i.e., if

only the sum of the concentrations for two or more metabolites is known, this information can be included in the model.

2.3 *A posteriori* analysis

The output of GRASP is a population of thermodynamically consistent models anchored at a reference state. This population is encoded as a MATLAB struct, which can be readily used to perform MCA, as well as time-course simulations using the functions provided. In addition, the user can export individual models to `SBML`. To visualize the MCA and simulation results, a small python package was developed and example Jupyter notebooks are provided in the GRASP repository.

The large-scale model provided as a tutorial is based on the *Pseudomonas putida* model in [?], which includes 79 reactions and GRASP takes ≈ 48 min to generate a model ensemble composed of 10,000 models on a laptop ASUS U36SG with an Intel(R) Core(TM) i5-2450M CPU @ 2.50GHz and 16GB of RAM when using a single core. For this task, Gurobi was used to solve the MILP problems of the thermodynamic formulation. Using the rejection sampler can be more computationally expensive though, depending on how many experimental conditions are supplied and how large is the specified maximum flux discrepancy between the model predictions and the experimental conditions. However, the software can take advantage of multiple cores, with an almost linear scaling. This is done by specifying the number of cores to be used in the GRASP input file. We provide several tutorials with the software in the Github repository and instructions on how to run them (`https://graspk.readthedocs.io`).

3 Optimization, sampling and numerical methods

Throughout the execution of the GRASP framework, different optimization formulations and sampling algorithms are implemented. In the following subsections, the latter are described in detail.

3.1 Modified TMFA

Thermodynamic-based Metabolic Flux Analysis (TMFA) [?] was employed to determine the feasible $\Delta G_i^{\mathrm{ref}}$ ranges for each metabolic reaction i in the set $(RXNS)$ of model reactions. Allowing for uncertainty in $\Delta_r G_i^{'0}$ used here to calculate $\Delta G_i^{\mathrm{ref}}$, we need to ensure that the sum of the $\Delta G_i^{\mathrm{ref}}$ for the reactions in any closed loop is zero. The modified TMFA formulation is presented as a MILP formulation:

$$
\begin{aligned}
\mathbf{S} \cdot \mathbf{v} &= \mathbf{0} && (6)\\
\Delta_r G_i^{\mathrm{ref}} - \Delta_r G_i^{'0} - RT \sum_{j=1}^{m} s_{i,j} \ln(x_j) &\leq \varepsilon\,,\ \forall i \in RXNS && (7)\\
-\Delta_r G_i^{\mathrm{ref}} + \Delta_r G_i^{'0} + RT \sum_{j=1}^{m} s_{i,j} \ln(x_j) &\leq \varepsilon\,,\ \forall i \in RXNS && (8)\\
-\mathbf{v} + K \cdot \mathbf{z} &\leq K \cdot \mathbf{1} && (9)\\
\mathbf{v} - K \cdot \mathbf{z} &\leq \mathbf{0} && (10)\\
\mathbf{\Delta_r G^{ref}} + K \cdot \mathbf{z} &\leq (K - \delta) \cdot \mathbf{1} && (11)\\
-\mathbf{\Delta_r G^{ref}} - K \cdot \mathbf{z} &\leq -\delta \cdot \mathbf{1} && (12)\\
\mathbf{N_{int}^T} \cdot \mathbf{\Delta_r G_{\mathrm{int}}^{ref}} &\leq \varepsilon \cdot \mathbf{1} && (13)\\
-\mathbf{N_{int}^T} \cdot \mathbf{\Delta_r G_{\mathrm{int}}^{ref}} &\leq \varepsilon \cdot \mathbf{1} && (14)
\end{aligned}
$$

where $\forall i \in RXNS$:

$$
\begin{aligned}
v_{i,\min} \leq v_i &\leq v_{i,\max}\\
\ln x_{j,\min} \leq \ln x_j &\leq \ln x_{j,\max}\\
-M \leq \Delta_r G_i^{\mathrm{ref}} &\leq M\\
\Delta_r G_{i,\min}^{'0} \leq \Delta_r G_i^{'0} &\leq \Delta_r G_{i,\max}^{'0}
\end{aligned}
$$

$\mathbf{S}$ is the stoichiometric matrix, $\mathbf{v}$ is the flux vector, v_i is the flux of each reaction i, $s_{i,j}$ is the stoichiometric coefficient for metabolite j in reaction i, $\Delta_r G_i^{\mathrm{ref}}$ is the Gibbs energy of reaction i at the reference state, $\Delta_r G_i^{'0}$ is the standard Gibbs free energy of reaction i (e.g., obtained from *eQuilibrator* [**?**]), x_j is the concentration of metabolite j, z_i is a binary variable which ensures that, for each reaction i, $\Delta_r G_i^{\mathrm{ref}}$ and v_i have opposite signs (i.e. the reactions are thermodynamically consistent), $\mathbf{N_{int}}$ is a null space of basis of the stoichiometric matrix of internal reactions $\mathbf{S_{int}}$, K and M are constants with arbitrarily high values (10^{12} and 10^5, respectively), ε is an arbitrarily low value (10^{-5} currently) used to deal with numerical imprecision, R is the gas constant in kJ/mol, and T the temperature in Kelvin (298.15 K). In Eqs. (13) and (14), $\mathbf{\Delta_r G_{\mathrm{int}}^{ref}}$ denotes the Gibbs energy vector of the internal reactions in $RXNS$.

Eq. (6) represents the steady-state condition, Eqs. (7)-(12) enforce the second law of thermodynamics, i.e. a flux v_i can only be positive if the respective $\Delta_r G_i^{\mathrm{ref}}$ is negative, Eqs. (13) and (14) ensure that for any closed loops (determined by $\mathbf{N_{int}}$) the sum of $\Delta_r G_{i,\mathrm{int}}^{\mathrm{ref}}$ for all reactions involved in a loop is zero.

The bounds $v_{\min}$, $v_{\max}$, $x_{\min}$, $x_{\max}$, $\Delta_r G_{i,\min}^{'0}$, and $\Delta_r G_{i,\max}^{'0}$ are determined from the input data. In general, the minimum value of a quantity will be its mean value minus two times its standard deviation, and the maximum value will be its mean values plus twice its standard deviation, i.e., $v_{\min} = v_{\mathrm{mean}} - 2\sigma_v$, and $v_{\max} = v_{\mathrm{mean}} + 2\sigma_v$.

By using the constraints and variable limits above, each variable is then maximized and minimized individually (using the MILP objective function) to determine the respective range that satisfies all the mass balance and thermodynamic constraints.

3.2 Flux and thermodynamic sampling

Given the bounds for each variable obtained from the modified TMFA, a variation of the general Hit-and-Run algorithm [?] is implemented to sample values for each variable within the determined bounds and that satisfy the constraints defined in TMFA. Since GRASP requires the directionalities to be consistent with the allowable flux ranges, thermodynamic quantities ($\mathbf{\Delta_r G}^{\text{ref}}$, $\mathbf{\Delta_r G^{'0}}$, and $\ln \mathbf{x}$) and fluxes $\mathbf{v}$ can be sampled separately. Importantly, in both cases the variables of interest are confined to convex regions which can be readily sampled using well-known efficient methods such as Hit-and-Run.

Steady-state fluxes are sampled consistent with the following constraints:

$$\mathbf{S} \cdot \mathbf{v} = \mathbf{0}$$

where:

$$\mathbf{v}_{\text{min}} \leq \mathbf{v} \leq \mathbf{v}_{\text{max}}$$

On the other hand, thermodynamic quantities ($\mathbf{\Delta_r G}^{\text{ref}}$, $\mathbf{\Delta_r G^{'0}}$, and $\ln \mathbf{x}$) are sampled consistent with these constraints:

$$\Delta_r G_i^{\text{ref}} - \Delta_r G_i^{'0} - RT \sum_{j=1}^{m} s_{i,j} \ln(x_j) \quad \leq \quad \varepsilon \,, \forall i \in RXNS \tag{15}$$

$$-\Delta_r G_i^{\text{ref}} + \Delta_r G_i^{'0} + RT \sum_{j=1}^{m} s_{i,j} \ln(x_j) \quad \leq \quad \varepsilon \,, \forall i \in RXNS \tag{16}$$

$$\mathbf{N}_{\text{int}}^{\mathbf{T}} \cdot \mathbf{\Delta_r G}_{\text{int}}^{\mathbf{ref}} \quad \leq \quad \varepsilon \cdot \mathbf{1} \tag{17}$$

$$-\mathbf{N}_{\text{int}}^{\mathbf{T}} \cdot \mathbf{\Delta_r G}_{\text{int}}^{\mathbf{ref}} \quad \leq \quad \varepsilon \cdot \mathbf{1} \tag{18}$$

where $\forall i \in RXNS$:

$$\Delta_r G_{i,\text{min}}^{\text{ref}} \leq \Delta_r G_i^{\text{ref}} \leq \Delta_r G_{i,\text{max}}^{\text{ref}}$$

$$\Delta_r G_{i,\text{min}}^{'0} \leq \Delta_r G_i^{'0} \leq \Delta_r G_{i,\text{max}}^{'0}$$

$$\ln x_{j,\text{min}} \leq \ln x_j \leq \ln x_{j,\text{max}}$$

3.3 MCA for promiscuous enzymes

Because we decompose each reaction into microscopic reactions and model each of them using mass-action kinetics, it is straightforward to model promiscuous enzymes. For instance, in some organisms, glucose-6-phosphate dehydrogenase (G6PDH) is a promiscuous enzyme that can use either NAD or NADP as cofactors. Assuming an ordered mechanism where NAD/NADP binds first and NADH/NADPH is released last, the following mechanisms of reaction can be written:

G6PDH + nad ⇔ G6PDH-nad
G6PDH-nad + g6p ⇔ G6PDH-nad-g6p
G6PDH-nad-g6p ⇔ G6PDH-nadh-6pgl
G6PDH-nadh-6pgl ⇔ G6PDH-nadh + 6pgl
G6PDH-nadh ⇔ G6PDH + nadh

G6PDH + nadp ⇔ G6PDH-nadp

G6PDH-nadp + g6p ⇔ G6PDH-nadp-g6p
G6PDH-nadp-g6p ⇔ G6PDH-nadph-6pgl
G6PDH-nadph-6pgl ⇔ G6PDH-nadph + 6pgl
G6PDH-nadph ⇔ G6PDH + nadph

where ENZYME-metabolite represents the complex formed by the enzyme and the metabolite, e.g., G6PDH-nad represents the complex formed by the enzyme G6PDH and the metabolite nad, g6p stands for glucose-6-phosphate and 6pgl is 6-phospho-D-glucono-1,5-lactone.

Notably, traditional Metabolic Control Analysis (MCA) assumes:

1. Additivity: a change in enzyme concentration leads to a proportional change in the respective reaction flux;
2. Independence: when the concentration of enzyme i changes, it only directly affects the flux of reaction i.

where the independence assumption is not satisfied with promiscuous enzymes, since a perturbation in the concentration of enzyme i can affect multiple reactions. Therefore, besides the traditional metabolic control analysis, a more general framework has been implemented for the calculation of flux and concentration response coefficients [**?**, **?**, **?**, **?**]:

$$\begin{aligned}\mathbf{R_e} &= \mathbf{C_v} \cdot \mathbf{\Pi} \\ \mathbf{R_x} &= \mathbf{C_x} \cdot \mathbf{\Pi}\end{aligned}$$

where $\mathbf{\Pi}$ is the parameter elasticity matrix, which contains the partial derivatives of each reaction w.r.t. each enzyme concentration,

$$\mathbf{\Pi} = \frac{\partial \mathbf{v}}{\partial \mathbf{p}}$$

$\mathbf{C_v}$ is the flux control coefficients matrix, calculated as:

$$\mathbf{C_v} = \mathbf{1} + \left(\frac{\partial \mathbf{v}}{\partial \mathbf{x}}\right) \mathbf{C_x} = \mathbf{1} + \mathbf{E} \cdot \mathbf{C_x}$$

where $\mathbf{E}$ is the concentration elasticity matrix and $\mathbf{C_x}$ is the concentration control coefficients matrix, calculated as:

$$\mathbf{C_x} = -\left(\mathbf{S} \cdot \mathbf{E}\right)^{-1} \mathbf{S} = -\mathbf{J_x}^{-1} \mathbf{S}$$

where $\mathbf{S}$ is the stoichiometric matrix and $\mathbf{J_x}$ is the Jacobian matrix.

References

[Chakrabarti *et al.*(2013)Chakrabarti, Miskovic, Soh, and Hatzimanikatis] Chakrabarti, A., Miskovic, L., Soh, K. C., and Hatzimanikatis, V. (2013). Towards kinetic modeling of genome-scale metabolic networks without sacrificing stoichiometric, thermodynamic and physiological constraints. *Biotechnology Journal*, **8**(9), 1043–1057.

[Gopalakrishnan *et al.*(2020)Gopalakrishnan, Dash, and Maranas] Gopalakrishnan, S., Dash, S., and Maranas, C. (2020). K-fit: An accelerated kinetic parameterization algorithm using steady-state fluxomic data. *Metabolic Engineering*, **61**, 197–205.

[Gurobi Optimization, LLC(2021)Gurobi Optimization, LLC] Gurobi Optimization, LLC (2021). Gurobi Optimizer Reference Manual.

[Gutenkunst *et al.*(2007)Gutenkunst, Waterfall, Casey, Brown, Myers, and Sethna] Gutenkunst, R. N., Waterfall, J. J., Casey, F. P., Brown, K. S., Myers, C. R., and Sethna, J. P. (2007). Universally sloppy parameter sensitivities in systems biology models. *PLOS Computational Biology*, **3**(10), 1–8.

[Kozaeva *et al.*(2021)Kozaeva, Volkova, Matos, Mezzina, Wulff, Volke, Nielsen, and Nikel] Kozaeva, E., Volkova, S., Matos, M. R., Mezzina, M. P., Wulff, T., Volke, D. C., Nielsen, L. K., and Nikel, P. I. (2021). Model-guided dynamic control of essential metabolic nodes boosts acetyl-coenzyme a–dependent bioproduction in rewired pseudomonas putida. *Metabolic Engineering*, **67**, 373–386.

[Saa and Nielsen(2015)Saa and Nielsen] Saa, P. and Nielsen, L. K. (2015). A general framework for thermodynamically consistent parameterization and efficient sampling of enzymatic reactions. *PLoS computational biology*, **11**(4), e1004195.

[Saa and Nielsen(2016)Saa and Nielsen] Saa, P. A. and Nielsen, L. K. (2016). Construction of feasible and accurate kinetic models of metabolism: A Bayesian approach. *Scientific Reports*, **6**(July), 29635.

[Saa and Nielsen(2017)Saa and Nielsen] Saa, P. A. and Nielsen, L. K. (2017). Formulation, construction and analysis of kinetic models of metabolism: A review of modelling frameworks. *Biotechnology Advances*, **35**(8), 981–1003.

[Steven G. Johnson(2021)Steven G. Johnson] Steven G. Johnson (2021). The NLopt nonlinear-optimization package.

[Villaverde *et al.*(2021)Villaverde, Pathirana, Fröhlich, Hasenauer, and Banga] Villaverde, A. F., Pathirana, D., Fröhlich, F., Hasenauer, J., and Banga, J. R. (2021). A protocol for dynamic model calibration. *Briefings in Bioinformatics*, **23**(1). bbab387.
